# Systematic content analysis: A combined method to analyze the literature on the daylighting (de-culverting) of urban streams

**DOI:** 10.1016/j.mex.2020.100984

**Published:** 2020-07-03

**Authors:** Luna Khirfan, Megan Peck, Niloofar Mohtat

**Affiliations:** The School of Planning, University of Waterloo, Ontario, Canada

**Keywords:** Systematic literature review, Content analysis, Stream daylighting, De-culverting, Urban streams, De-culverting, Waterways

## Abstract

In this era of climate change, novel nature-based solutions, like the daylighting (de-culverting) of streams, that enhance the socio-ecological resilience are gaining prominence. Yet, the growing body of literature on stream daylighting spreads over an array of seemingly disconnected disciplines and lacks consistency in the terminology and the definitions of the practice. Moreover, nearly all the literature review studies on stream daylighting (mostly produced since 2000) underscore, as their point of departure, the daylighting projects rather than a review of the literature's content per se. Therefore, this study reassesses the literature on stream daylighting with a particular focus on its role, as a nature-based solution, for climate change mitigation and adaptation and for socio-environmental justice. We combine the systematic literature review (an all-encompassing review of the available literature on stream daylighting) with the inductive content analysis (an in-depth analysis of this literature's nature). Accordingly, we investigate all the relevant English-language publications since the first peer reviewed article on stream daylighting was published in 1992 until the end of 2018 to analyze four themes: the disciplines and sub-disciplines of the literature; the terminologies and synonyms of stream daylighting; the definitions of stream daylighting; and the case studies tackled in the literature.

• We develop a method that combines a systematic review of the stream daylighting literature and inductive content analysis.
• The method provides insights on the stream daylighting’s literature’s disciplines, terminologies, synonyms and case studies.
• The method is adaptable particularly, to nascent areas of study where sources’ numbers range between 100-200.

## Graphic abstract

**Figure:**
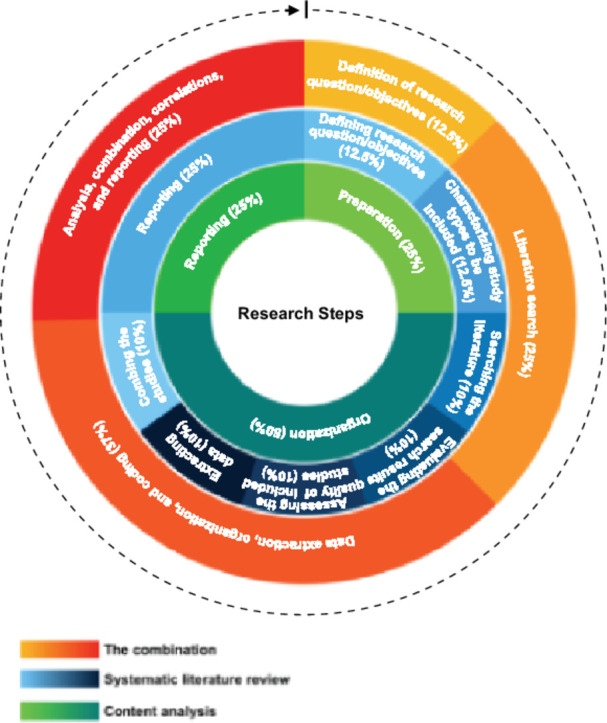

**Table:** Specifications Table

Subject AREA	Environmental Science
More specific subject area	Nature-based solutions, specifically, stream daylighting (i.e., de-culverting buried streams)
Method name	A combination of a systematic literature review method and an inductive content analysis
Name and reference of original method	A combination of two methods:
	1) Systematic literature review [29,32]
	2) Content analysis [13]
Resource availability	Two resources (raw data and website)
	https://uwaterloo.ca/stream-daylighting/literature-review-database
	https://uwaterloo.ca/stream-daylighting/interactive-map

## Background

There is no agreement on the earliest daylighting because the re-naturalization of the riverbed is considered an important criterion by some, but not necessarily so by others who consider any de-culverting initiative, even if in an open channel, to be daylighting. Accordingly, the first daylighting project could either be Napa Creek in Napa Valley, California (completed in the 1970s), which de-culverted in an open channel or Strawberry Creek in Berkeley, California (in 1987), which was de-culverted and re-naturalized [8,33,34]. Almost concurrently with Strawberry Creek, the city of Zürich, Switzerland's initiated its daylighting policy in the 1980s that led to the first implementation in 1988 and eventually to a network of over 21 km of daylighted brooks [10], [14]. Regardless of which project is considered the first, the practice of stream daylighting came to the forefront in 2004, after the completion of the nearly US $281 million Cheonggyecheon stream daylighting project in downtown Seoul, South Korea [23].

Thus, not only does the definition of what constitutes stream daylighting vary, but also, the literature on the subject seems to be multi-disciplinary in nature – tackling the topic through completely different disciplinary lenses. These variations are probably attributed to the relative nascence and novelty of the daylighting literature and the diversity of the implemented daylighting projects in respect to their nature, scale, size, costs, and outcomes, among others. This prompted this study which comprehensively reviews, interprets, and combines the scientific and the grey literature on stream daylighting in order to better understand its evolution, nature, and scope.

## The methodological bases: systematic literature reviews and content analysis

Literature reviews are primarily qualitative synthesis that provide critical tools for understanding a topic's discourse especially, in a climate of increasing, and often deviant and contradictory, research output [36]. Literature reviews serve as a research-orienting device to identify trends, gaps, intersections, directions or issues within the broader research scope, hence, inform and ground future research trajectories [11,36]. Considered important scholarly contributions, literature reviews help to map, consolidate, synthesize and refine scattered knowledge in a given field and springboard theory development. The major critiques of literature reviews (relative to rigorous, objective, and replicable empirical studies) are a lack of stringent procedures and the reliance on subjective interpretations by (experienced) scholars [36]. Consequently, systematic and transparent processes for reviewing the literature emerged, in which the analysis is independent of researchers’ bias where methods of systematic categorization, such as content analysis, provide rigorous, systematic, and replicable methodological frameworks [17,36]. This research, therefore, combines the two methods: the content analysis and the systematic literature review. The next sections discuss these two methods; present their novel combination for this study; and their individual limitations as well as the advantages of their combination.

### What is systematic literature review and how has it been traditionally applied?

Researchers often need reviews to arrange and give priority to the existing knowledge. In this respect, the systematic literature review emerged as a new way of reviewing the literature [31]. The systematic literature review as an accurate, thorough, and rule-guided method helps the researchers to understand large amounts of information by discovering, assessing, analysing, and combining the existing data regarding a specific research question. With that being said, systematic reviews not only present useful information but also evidence-based solutions [3]; in other words, systematic reviews facilitate distinguishing authentic knowledge from assumed knowledge [31]. Although widely recognized in health research, systematic literature reviews are less used in the social sciences [15]. Currently, no studies exist in the ISI Web of Knowledge that use this method to study stream daylighting (de-culverting), and just 11 papers systematically review the literature on any account of stream restoration (using the consecutive search keywords and Boolean operators: ‘stream daylighting’ AND ”systematic review” OR ”systematic literature review”; AND ”stream restoration” AND ‘”systematic review” OR “systematic literature review”− see: [20]: 2).

Systematic literature reviews are different from traditional reviews in terms of three methodological approaches: 1) they start with delineating a research strategy; 2) they identify explicit criteria for including and/or excluding the literature; and 3) they seek to gather, evaluate, and interpret as much available and relevant literature as possible [15]. These approaches overcome the shortcomings of the generality of a traditional review in both scope (i.e., number of articles reviewed) and nature (i.e., un-structured protocol and reduced replicability) which, because of bias, may produce inconsistencies among reviews of the same topic [27].

### What is content analysis and how has it been traditionally used?

Content analysis is an objective, rule-guided technique used to make replicable and valid inferences by analysing (coding) the characteristics of visual, verbal, and/or written documents [13,16,17]. Through systematic evaluation, qualitative data can be converted into quantitative analysis, which is used to increase the methodological rigor of literature reviews [16]. This transparent framework is applied for the purpose of describing or evaluating a subject to provide new insights, understandings, interpretations and, consequently, a guide for action [13,22]. The limitless disciplinary application of content analysis led to its application in various fields including, anthropology, political science, psychology, and business to name but a few [13,28].

Content analysis can take on a quantitative and/or qualitative approach, applied either inductively or deductively depending on the specific research questions and research design [13]. Broadly speaking, this method incorporates two analysis levels [36]. The first level includes analysing the manifest content of the text through statistical means. This is often viewed as a *quantitative* approach, where classification is predominantly pre-determined and deductive, with the data analyzed statistically [16]. However, this method of analysis has been criticized for being over simplified and distorted in meaning [25]. Resultantly, the second level of analysis involves interpreting the manifest and latent content of the text, facilitating, through rigorous analyses, an understanding of a phenomenon's critical processes, motives and objectives, while deriving rich meanings and insights from the text [9,12,13,16,36,37]. Under this *qualitative* structure, data can be categorized deductively or inductively (based on the research question) and applied by a close reading of the text [9,16].

Content analysis shares similarities with other forms of data analysis, in some cases overlapping with other methods to a considerable extent, including coding, thematic analysis and grounded theory. These methods share a conceptual process of data abstraction, whereby a hierarchical coding form is applied, and categories or codes are assigned to segments of the data [35]. But in other respects, the objectives and, or procedures of these methods are different - selected based on the specificities of the research question [9,35,37].

To begin with, ‘coding’ may refer to a variety of different procedures, but in general terms, it involves an analytic and iterative procedure, whereby categories are data driven, materials are varied and diverse, and categorical consistency (i.e., systemization) is less of a concern, hence, the process is more descriptive than analytic. Alternatively, qualitative content analysis consists of a more linear, descriptive process based on a focused material scope (e.g., textural documents only), where categories are concept-driven, and consistency is an important criterion. Accordingly, qualitative content analysis is appropriately used for descriptive research questions, rather than those that are analytic in nature [35]. As for thematic analysis and grounded theory, they seek, similarly to content analysis, to better understand a phenomenon by searching text for patterns and themes; however, they differ in the specific research objectives and, consequently, in the application of their research methods. While both content analysis and thematic analysis target a relatively low level of interpretation, the focus in grounded theory is on substantive theory development (to explain a phenomenon in a given context) –entailing a higher level of interpretive complexity [37]. Yet, content analysis lends itself better to more generalizable, descriptive research questions than thematic analysis [9,35]. Moreover, although the three approaches deploy the consistent comparative analysis, they do so differently. Specifically, thematic analysis provides a purely qualitative account of the data while content analysis facilitates both quantitative and qualitative data analysis [37]. Also, a strictly iterative process of concurrent data collection and theoretical sampling is applied in grounded theory to investigate the connections between and among categories. In contrast, because qualitative content analysis seeks to extract categories from the data, it is therefore more flexible and employs inductive and/or deductive processes [9].

### How does a systematic literature review fit with this article's research objectives/approach?

The first peer reviewed publication on stream daylighting from 1992 is Charbonneau and Resh's study of Strawberry Creek [8], which was published in Aquatic Conservation: Marine and Freshwater Ecosystems. Since then, all that may be considered “literature reviews” on stream daylighting were predominantly produced in the first decade of the 21st Century. Starting in 2000 with Pinkham's [33] review of stream daylighting projects; followed by Buchholz and Younos's [6] evaluations of case studies; Wild et al.’s [39] assessment of the objectives and the benefits of implemented projects globally; and last, Broadhead and Learner's [4] online database of daylighting projects and Broadhead et al.'s [5] global review of the streams captured in combined sewers. Yet, the departure point for all these reviews stems from a focus on the stream daylighting projects rather than on a review of the literature per se. Moreover, and considering that nowadays, vast amounts of information are produced in short periods of time, 34 years after stream daylighting's first appearance in the literature (see: [8]), a systematic review is warranted in order to manage and synthesize the literature and update researchers on the trajectory of the discipline. In addition, considering that nearly nine years have passed since Wild et al.'s [39] review of stream daylighting projects^1^ and seven years since Broadhead and Learner's [4] online database of daylighting projects, it is crucial to revaluate the literature on the subject. The shift in environmental perspectives also brings to the fore the significance of stream daylighting as a nature-based solution for mitigating greenhouse gases, adapting to climate change, and advancing socio-environmental justice. Accordingly, a systematic literature review at a global scale becomes essential to characterize and identify the studies that provide an understanding of stream daylighting's nature – which calls for an all-encompassing review of as much as possible of the available literature.

### How does content analysis fit with our research objective/approach?

Content analysis is an appropriate research method for gaining a stronger grasp of the nature and scope of the literature on stream daylighting. This study applies qualitative content analysis to analyze the literature on the daylighting of urban streams whereby, for the purposes of this study, we define qualitative content analysis as: a tool that subjectively determines the presence of specific words, ideas, themes, and/or concepts within particular qualitative data (i.e., text in this case) through systematic classification or coding [9,13,36]. Such a study framework enables the researchers to qualify and analyze the existence, meanings, and connections among certain words, themes, and/or concepts in order to develop generalizable inferences [13,37]. As a novel and relatively new topic, it was not until 1992 that stream daylighting was first mentioned in the academic literature (see: [8]). Since then, empirical studies on this practice have been limited and fragmented over multiple disciplines, thus warranting an exploratory research study with an inductive approach that facilitates the development of categories through qualitative content analysis for all the studies published between 1992 and 2018 (see: [13,35,37]). Therefore, content analysis suits this study's focus, which is to systematically describe both the manifest and latent content of the literature related to stream daylighting rather than develop theory [9]. Additionally, content analysis allows for a framework that incorporates both quantitative and qualitative evaluations [37].

## Methodology: a combined research method to delve into the latent and manifest content of the literature

We combined the systematic literature review and content analysis to identify the evolution, nature, and scope of the literature on stream daylighting. Specifically, the systematic literature review consists of eight steps, namely: 1) defining the research objectives and their associated questions; 2) determining the types of publications that need to be included in the review; 3) searching the literature; 4) evaluating the search results; 5) assessing the quality of the sources included; 6) extracting the data; 7) combing the sources; and lastly, 8) reporting [29,32]. As for the inductive content analysis, it takes place in three broad steps: 1) preparation; 2) organization; and 3) reporting [13]. Our combination led to a methodological framework consisting of four distinct steps, namely: (1) delineating the objectives and the questions of the research project; (2) searching the literature; (3) extracting, organizing, and coding the data; and (4) analysing, combining, correlating, and reporting the data (refer to the graphical abstract for details) [20].

### Step I: research questions and preparation

#### Delineating the objectives/questions of the research (systematic literature review)

We began with the formulation of the research questions based on the review's objectives, namely: how did the discourse on stream daylighting evolve? And what is the scope and nature of this discourse? Specifically, as discussed in Khirfan, Peck, and Mohtat [20], we investigate four important themes in reviewing the literature, namely: (sub)disciplines, terminologies, definitions, and case studies. We, specifically, identify the relationships and correlations among the themes by exploring the following research questions (see [31] on formulating questions):

1. Disciplines:
  a. How has the stream daylighting discourse appeared and evolved during time?
  b. How does the discourse connect with the locations of sources’ authorship (continent and country)?
  c. In which disciplines and sub-disciplines is stream daylighting tackled?
  d. What is the interdisciplinary nature of the discourse? And What can we learn from analysing its multidisciplinary nature?
2. Terminologies:
  a. How does the literature describe stream daylighting? which synonyms/terminologies does the literature use for describing stream daylighting?
  b. How have stream daylighting's terminologies transformed and evolved during time?
  c. How are the uses of terminologies connected with the publication types of sources(i.e., grey literature versus peer reviewed) and with the authorship locations of the sources (i.e., continent and country)?
3. Definitions:
  a. How does the literature define stream daylighting? And what definition tracks can be extracted from the literature?
  b. How have the definitions altered during time?
  c. How are the uses of definitions connected with the publication types of sources (peer-reviewed versus grey literature) and with the authorship locations of sources (i.e., continent and country)?
4. Case studies:
  a. What are stream daylighting case studies that the literature cite the most?
  b. In which country and continent does these most cited case studies located? how does the distribution of each case study around the world connect with its citation frequency in the literature?
  c. Why and how does the number of sources that cite each of the case studies change during time?
  d. How is the number of sources that cite each of the case studies associated with the sources’ authoring location (country and continent)?
5. Also, we examine the relationships among the four above-mentioned themes, particularly:
  a. How do the stream daylighting's terminologies connected with the definition tracks and with the case studies cited within the sources?
  b. How do the literatures’ disciplines correlated with stream daylighting's terminologies, definition tracks, and case studies that are mentioned within the literature?

Lastly, building on the answers, we identify the gaps that exist in the current studies and the trajectories that the literature need to adopt in future (on these, refer to [20]).

#### Preparation (content analysis)

Preparation, also known as the material collection phase, entails identifying the unit of analysis [9,25] which may range from part of, or all of, a textual document [13,36]. During the preparation phase, formal characteristics of the material (e.g., publisher, publication year, author location & affiliations etc.) are also assessed so as to provide the background for the subsequent descriptive analyses [25,36].This study commenced with a broad unit of analysis, namely, English-speaking peer reviewed sources on stream daylighting, which was further refined in the second step of the study with additional criteria.

### Step II: searching the literature (combined systematic reviews and content analysis)

This step entailed an iterative four-task process consisting of: identifying the sources, characterizing their types, assessing their quality, evaluating the search results, and repeating.

Once the actual search for the sources commenced (i.e., *identifying the sources*), the research team used keywords like “stream daylighting”, “de-culverting” within three academic databases namely: Primo, Google, and Google Scholar (on carrying out database searches, see: [29]). An initial overview of the peer reviewed sources gathered at this phase led to several observations with direct implications on our research project (i.e., characterizing the sources’ types and assessing their quality). To begin with, the relative nascence and uptake of the stream daylighting practice (compared to other nature-based solutions) ensued in a limited quantity of the peer reviewed literature on the topic. Also, we identified the “Cheonggyecheon” project in Seoul (South Korea) as a world-renowned stream daylighting project and the City of Zürich's (Switzerland) policy as unique in the world, which led us to include the keywords “Cheonggyecheon” and “Zürich” in our search of the databases. Moreover, we observed from our careful reading of the contents of the sources that: a) the Cheonggyecheon was referred to by the term ‘restoration’ rather than ‘daylighting’, and that b) some sources discussing cases other than the Cheonggyecheon also often deployed terms synonymous to, but not ‘daylighting’ per se.

Thus, after *evaluating the search results* a decision was made early on in the research design to refine the unit of analysis so as to include, in addition to peer-reviewed English sources, the grey literature, in reference to non-peer reviewed sources, including: books, book chapters, governmental reports, conference articles and proceedings, and undergraduate and graduate students’ theses and Ph.D. dissertations. As Adams, Smart, and Sigismund Huff [1] assert, the incorporation of grey literature within systematic literature reviews expands the domain of research evidence to incorporate those studies that are omitted in the academic literature, which is specifically relevant for the relatively new stream daylighting subject matter. Such inclusion also expands the audience of the research to cover both practitioners and academics [1]. Moreover, we, for the purpose of broadening the range of the included studies, carefully investigated the references of each source in order to include the ones relevant to stream daylighting. For these sources, we first consulted the title, abstract and keywords and if it was not possible to determine their relevance to this study, we made a decision to read the entire text.

**Fig. 1:**
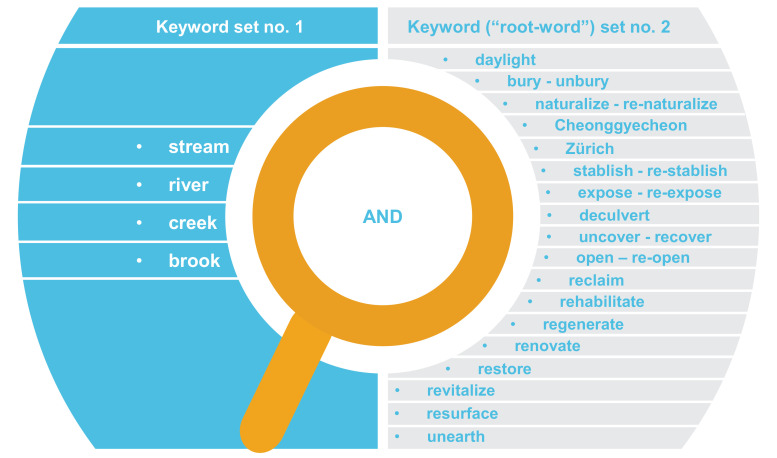
The keywords we used in our Boolean search (also refer to: [20]: 3).

Furthermore, we conducted a careful reading of the contents of the sources to gradually, iteratively, and systematically document all the synonymous terms used for ‘daylighting’ and included them in our search keywords along with ‘Cheonggyecheon’ (see Fig. 1) on the keywords used in the search). Although time and labor intensive (extending between June 2017 and December 2018), this process ensured that we include as many as possible (if not all) of the sources relevant to stream daylighting. Eventually, a total of 118 sources were identified, of which 81 are peer reviewed sources and 37 are grey literature sources (see Table 1) [20].

**Table 1:** The types of the sources identified.

Types of sources	Total	The root-term “Daylight” mentioned directly	The root-term “daylight” not mentioned directly, synonymous terms are used	The root-term “daylight” and/or its synonyms are not mentioned, but the source discusses the burying (culverting, not daylighting) of streams
I. Peer-reviewed journal articles	**81**	**50**	**27**	**4**
1. Journal articles	78	48	26	4
2. Book reviews	3	2	1	0
II. Books (edited books)	**8**	**6**	**2**	**0**
III. Book chapters	**1**	**1**	**0**	**0**
IV. Institutional reports	**11**	**9**	**2**	**0**
V. Conference papers	**1**	**1**	**0**	**0**
VI. Student work	**15**	**14**	**1**	**0**
1. Student reports	5	4	1	0
2. Undergraduate thesis	1	1	0	0
3. Master's thesis	8	8	0	0
4. Doctoral dissertation	1	1	0	0
VII. Workshop proceedings	**1**	**1**	**0**	**0**
Total	**118**	**82**	**32**	**4**

### Step III data extraction, organization, and coding (combined systematic literature review and content analysis)

After determining all the studies that should be included, we extracted, in the third step, the relevant information from each source, including the manifest (formal) and latent content. ‘*Manifest*’ data, which may be directly measured or observed, presents those facts about the literature that are easily accessible and indisputable while, in contrast, ‘*latent’* data represents information that is not directly observable or measurable, and which otherwise requires careful reading of each source (see [29] on these two types of data).

The relevant ‘*manifest*’ data of the sources included: the types of publications (i.e., peer reviewed versus grey literature); SJR's^2^ disciplinary classification; the date of publication; and the geographic location of the authorship (continent/country). For example, in order to analyze the multidisciplinary and transdisciplinary nature of the peer-reviewed literature, we organized the resulting disciplinary ‘subject areas’ under one of the following knowledge categories: 1) natural science; 2) social science; or 3) arts and humanities. For the purposes of this study, we consider a journal as multidisciplinary when it features two or more ‘subject areas’ in SJR's database (as opposed to transdisciplinary when an article transcends its traditional disciplinary boundary by integrating more than one branch of knowledge based on the three categories delineated above). The manifest data were recorded in a Microsoft Excel database.

Extracting the *‘latent’* content entailed carefully reading each source in order to identify: 1) the terms that are synonymous with stream daylighting (which led to the terminology analysis); 2) the different definitions of stream daylighting (which we categorized under the definition tracks); and 3) the stream daylighting case studies and projects mentioned in each of the sources (which led to the analysis of stream daylighting case studies/projects). A reductionist process included descriptive analytical assessments of the collected sources that led to their ‘organization’ into categories (i.e., ‘descriptive analysis’) (see [9,13,25]). Categories refer to items “with similar meaning and connotations” that could either be mutually exclusive or collectively exhaustive [9,38]. This categorization employs an open coding process which, in a literal form, entails writing headings and notes in the margins when reading the text then transcribing these headings onto a coding sheet, where categories can be freely generated and consolidated [13]. In order to avoid subjectivity and researchers’ bias, and so as to ensure replicability, we operationalized this approach by organizing our notes as an annotated bibliography in a Microsoft Word document which delineated the sources’ nature (unit of analysis) and content (how they address stream daylighting) (see the samples provided in Table 2). Accordingly, the information extracted from each source was organized into headings that, typical of an inductive approach, emerged from the latent content of each source, rather than from preconceived groupings, theories, or notions.

**Table 2:** Sample from organizing the annotated bibliography document into a standardized format to facilitate the process of categorization of the latent content.

**Charbonneau and H. Resh, 1992 – Strawberry Creek on the University of California, Berkeley Campus: A case history of urban stream restoration**	
**Keywords:** Restoration; Environmental education; University of California; Ecological, Urban development; Strawberry Creek	
**Article summary**	**Terminology**
The article investigates the history of covering strawberry creek and its restoration impacts.	**Restore**
	• The term “restore” is used dominantly for describing the project.
	• The authors have used the root-term “restore” for describing the uncovering process of Strawberry creek for the purpose of reducing the level of pollutants and erosion.
	**Rehabilitate**
	The root-term “rehabilitate” is used as a synonym for “restoration” in this article.
	**Definition**
	The paper has not offered any definition for stream daylighting.
	**Case study**
	**Strawberry creek (North America; USA):**
	• The restoration of Strawberry creek has many educational benefits for students in the University of California. This restored creek is used for laboratory exercises for students. .
	• After the restoration of Strawberry Creek, the amount of nutrient and bacteria in downstream dramatically reduced.
	The restoration of Strawberry Creek has had aesthetic benefits for University of California, Berkley campus.

**Moran et al., 2016 – Finding our way: A case study of urban waterway restoration and participatory process**	
**Keywords:** Creek, Restoration, Participation, low-income people, Co-production, Environmental Justice, Revitalization, Rehabilitation, Socioeconomic	
**Article summary**	**Terminology**
This study delves into the challenges of using collaborative participatory approach in Onondaga creek restoration in Syracuse, New York. The study concludes that the project faced three challenges: (1) achieving environmental justice ideals with participation of the communities; (2) explaining technical issues in a clear way for local people; (3) reaching to a consensus amongst the community.	**Restore**
	• The authors have used the root-term “restore” to discuss about bio-physical, hydrological, habitual, and aquatic aspects of streams.
	**Revitalize**
	The root-term “revitalize” is used both for bio-physical aspects of streams and for broader socio-economic dimensions.
	**Definition**
	The paper has not offered any definition for stream daylighting.
	**Case study**
	The article has not mentioned any stream daylighting case study. Noteworthy to mention that the restoration project of **Onondaga Creek** is discussed. However, this project is a de-channelization project, not a de-culverting and/or stream daylighting project.
**Arango et al., 2017 – Urban infrastructure influences dissolved organic matter quality and bacterial metabolism in an urban stream network**	
**Keywords:** Buried streams, daylighting, dissolved organic matter fluorescence, extracellular enzyme activity, nutrient diffusing substrata	
**Article summary**	**Terminology**
The article investigates seasonal changes in dissolved organic matter (DOM) in three buried and open streams (in Cincinnati, OH). The results show that DOM have a higher quality in spring in comparison to other seasons. Moreover, humic DOM is higher in open sections of streams.	**Daylight**
	• The paper uses the term “stream daylighting” for suggesting a way for the management and restoration of urban streams.
	**Restore**
	• The root-term “restore” is used as “daylight” synonym.
	**Definition**
	**Engineering** (The inductive content analysis, later, organized this heading under “engineered-resilience” definition track -see Table 3)
	• "Stream daylighting is an engineering approach to urban stream restoration whereby buried streams are redesigned to be open to light".
	**Case study**
	The paper has not mentioned any stream daylighting project. It has just discussed about three anonymous buried streams in Cincinnati, OH (U.S.A.).

### Step IV: analysis, combination, correlations, and reporting (combined systematic reviews and content analysis)

Following the data extraction-organization-coding process, the analysis, combination, correlations followed during which we evaluated the heterogeneity of the data, combined the manifest data and latent content of the literature, and identified patterns and/or gaps within the literature. Accordingly, we analyzed trends in the literature by means of both quantitative (descriptive statistics) and qualitative (narrative review) approaches that assured objectivity and replicability (see [7] on this important combination). To achieve this, we created a Microsoft Excel database that combined the manifest and latent content (Table 3). While including the manifest content is straightforward, the coding of the latent content required following a protocol that ensured rigour. To elaborate, the headings that emerged in the previous step served as cross-cutting themes that were further organized, during this analysis step, into groupings of categories that were subsequently coded in the Microsoft Excel coding sheet (along with the manifest data), where it was possible to freely generate categories by consolidating, collapsing, or splitting groupings (see samples in Tables 2 and 3) (on coding, see: [13]). For example, with regards to the definition of stream daylighting, the researchers thoroughly read each source and whenever a definition of stream daylighting was offered, this was carefully documented in the annotated bibliography. Of the total 118 sources, 40 explicitly defined stream daylighting. Based on their nature, these definitions were grouped under general headings and gradually refined into a category scheme through continued deduction, interpretation and abstraction of the underlying meanings of the applied definitions.

**Table 3:** Sample of the Microsoft Excel database (showing the same four references in Table 2).

Manifest data							Latent data		
Publication Title	Authors	Publication year	Authorship location	Publication type	SJR discipline	SJR Sub-discipline	“Daylighting” terminology and/or its synonyms	Definition track	Case study location
“Strawberry Creek on the University of California; Berkeley Campus: A case history of urban stream restoration”	Charbonneau and Resh	1992	North America (USA)	Journal article	Agricultural and biological sciences; Environmental science	Aquatic science; Ecology; Nature and landscape conservation	restore; rehabilitate	N/A	North America (USA)
“Finding our way: A case study of urban waterway restoration and participatory process”	Moran et al.	2016	North America (USA)	Journal article	Environmental science	Ecology; Management; Monitoring; Policy and law; Nature and landscape conservation	restore; revitalize	N/A	N/A
“Urban infrastructure influences dissolved organic matter quality and bacterial metabolism in an urban stream network”	Arango et al.	2017	North America (USA)	Journal article	Agricultural and biological sciences	Aquatic science	daylight; restore	engineering resilience	N/A
“The frog dilemma: urban stream restoration and the nature/culture dialectic”	Newman et al.	2012	North America (Canada)	Journal article	N/A	N/A	daylight; restore; revitalize; rehabilitate	De-culverting	North America (Canada); Asia (South Korea)

The analysis, combination, and correlations also entailed the judicious use of computer assistance was used to facilitate the identification of data trends and relationships. Accordingly, and in order to prepare data records of the Microsoft Excel database for analysis, we used Alteryx software (for cleaning and organizing the data) and Tableau software (for visualizing the data). It is essential to note that a similar column (e.g., the column of the ‘authors’ of all the sources) connects the Excel and the Tableau datasets to facilitate relational data querying, and to improve the recognition of mutual patterns that exist in the literature [20].

Lastly, during reporting, which is also known as ‘*material evaluation’*, the emergent themes from the latent content were established as a means to link underlying meanings and concepts (see [36] on this process). The trustworthiness of the resultant data is also typically examined during this phase, including the triad of credibility (i.e., being trusted), transferability (i.e., being applicable), and dependability (i.e., being consist) (see: [9,13]). To advance credibility, our research team adopted several approaches throughout the research project's lifespan including, member checking, peer debriefing, and showing representative quotations whereby individual members of the research team checked the themes independently then, through team debriefing, verified the findings (see [9] and [13] on these strategies). To facilitate transferability, this MethodsX article establishes the context of study by providing the details of data collection, management, and analysis in order to allow comparisons to be made (see: [9,13]). Also, following Cho and Lee's [9] recommendation, our research team ensured an audit trail, including carefully: recording all the study's steps, detailing notes on methodology, and documenting all the sources’ records, annotations, classifications, and eventually, themes (see: [9,13]).

## Individual limitations versus advantageous combination

### What are the advantages and disadvantages of systematic literature reviews?

The systematic nature and broad scope of systematic literature reviews move beyond superficial inspection into the applied understanding of reality. In doing so, this methodology is advantageous for its ability to summarize vast amounts of data and ideas for absorption [26], but simultaneously, the enlarged data sets of systematic reviews may yield copious sources [19] whose analysis can be time consuming and resource intensive. Due to the relatively small dataset in this study, however, it was possible to overcome this disadvantage.

Another challenge in systematic literature reviews pertains to inconsistencies in interpreting the inclusion and exclusion criteria among the different team members [24]. To combat these limitations, our team devised a clear, systematic review protocol which was followed strictly by each member of the research team, which ensured that all team members remain ‘on course’ throughout this study's steps, which ensured dependability (consistency). Additionally, the research team preformed team-debriefing, member checking, and ensured the production of an audit trail that facilitated consistency across researchers to verify findings and improve credibility and, by consequence, to ensure replicability.

### What are the advantages and disadvantages of content analysis?

Content analysis is advantageous for understanding the social reality of multifaceted, sensitive phenomenon [9] like stream daylighting, which overlaps urban ecology, urban planning, and landscape architecture among others. Content analysis is apt for analysing large volumes of textual data from varying sources, which facilitates corroborating evidence in exploratory or descriptive studies [2,9,13]. Additionally, because it facilitates the synthesis of large quantities of dynamic information, content analysis provides the means to study data retrospectively, which enables tracking changes to processes and trends over time ([2]: 139, [21]). This leads to an additional advantage, namely the benefit to conduct subsequent content analyses on the same subject at later points in time [21]. Furthermore, this method has been praised for its ability to combine both quantitative and qualitative approaches in order to retain rich meaning, supported by rigorous analyses [12,13]. When used correctly, qualitative content analysis provides a transparent research framework, providing researchers with clear, replicable and user-friendly methods for analysing data [37].

The literature outlines three primary critiques of content analysis: 1) inconsistency in its definition and procedural framework; 2) ineffectiveness at testing causal relationships; and 3) labor and time intensive nature (on these limitations, see: [2,9,16,18,21,37]). To overcome the first two limitations, parallels and distinctions of alternative research methods (grounded theory and thematic analysis) were reviewed and situations warranting the appropriate use of each method were presented (refer to Section “What is content analysis and how has it been traditionally used?”). Furthermore, it is acknowledged that the output of this study does not denote causal relationships, but rather the magnitude and, or patterns of certain responses. Last, the careful reading of the sources was indeed labor intensive (extending between June 2017 and December 2018), yet, the experience provided the research team members with the benefit of in-depth knowledge of the literature's content.

More important, our combined methodology exploits the strengths of each method. Consequently, our method has a clear procedure to collect and evaluate as much relevant literature available on stream daylighting as possible while deploying clear and replicable criteria for including and excluding sources [15]. Moreover, the structured and rigorous method for the extraction and the analysis of both the manifest and latent data through discovering, assessing, analysing, and combining the data [32] along with the structured strategies of qualitative content analysis reduce likelihood of research bias (hence, improve objectivity) and increase reliability and replicability [13,27,30].

## Additional information

Link to the research project's website: https://uwaterloo.ca/stream-daylighting/.

Link to the interactive database of this study: https://uwaterloo.ca/stream-daylighting/literature-review-database.

Link to the interactive map of daylighting projects around the globe: https://uwaterloo.ca/stream-daylighting/interactive-map.

## Footnotes

1 This peer reviewed journal article reviews all the daylighting projects globally.

2 SJR is an abbreviation for Scopus's SCImago Journal Rank which ranks academic journals factoring both the number of citations and the journal's impact whereby a higher SJR value indicates a higher prestige. This online ranking database categorizes the disciplinary ‘*subject area’* and the ‘*category’* of peer reviewed and academic journals. SJR encompasses the ISI's Web of knowledge database because of the broad journals which it covers. Note that, in SJR classification system, one journal might tackle multiple sub/disciplinary classifications [20].
